# The Nursing Supplement

**Published:** 1889-12-07

**Authors:** 


*The Hospital,*
DECEMBER 7, 1889.

*Extra Supplement*

### The Nursing Mirror.

All Contributions for this Supplement should be addressed to the Editor, THE HOSPITAL, 139-140, Salisbury Court, Fleet Street, London, E.C., and should have the word "Nursing" plainly written in left-hand top corner of the envelope.

### En Passant.

INCORPORATION OF THE MIDWIVE'S INSTITUTE.—An important point has been scored by the Midwive's Institute, of 15, Buckingham-street, Strand. They have secured a charter, which we hope is a first step towards registration, and they have got a reserve fund of £1,000. The members are becoming more and more numerous, and at the late committee meeting it was resolved that steps must be taken to open the club daily to all midwives and nurses who are members. Once more we urge upon all our readers who are versed in midwifery to join the institute, and strengthen its hands by every means in their power. The registration scheme it promulgates is solely for midwives, and does not identify these members with nurses—a move made by one society, but found to be as unpopular as it is impracticable and impolitic.

MARRIAGE AND THE PENSION FUND.—It is a universal error that it is not necessary in youth to save for old age. Women live cheerfully trusting that "the good man" will come along in time and that they will be provided for for the rest of their lives. But "the good man," as a rule, is a very sordid individual, and he prefers a wife who is thrifty and who has a nest-egg of her own. Therefore, though to all nurses may come the chance of marriage, that chance is not altered by the fact that she has saved some small sum of money; for she will have given proof of her thriftiness, and will have a dower. To discourage reckless and hasty marriages, some of the hospitals provide in their scheme of affiliation that sisters or nurses withdrawing their own half of the premiums deposited with the National Pension Fund whilst in the service of the hospital, or within twelve months after leaving, will forfeit the amount paid to their name by the hospital.

ASYLUM ATTENDANTS.—We are very sorry that our asylum readers have sent no dolls to the exhibition: they seem to rather lack the "go" of their hospital sisters. Perhaps this is because their hours of labour are generally longer—from 6 a.m. to 8 p.m. at Banstead Asylum, with the day off every fortnight, and ten days holiday in the summer. The wages of asylum attendants varies considerably: at Montrose the head female attendant gets £35, the ordinary attendants £20 a year, the matron gets £80. Very often the attendants are given a Christmas gift if their year's record has been good. With regard to fracture of the ribs, a frequent accident with the insane, Dr. James C. Howdon says: "Fracture of the ribs usually happens to general paralytics, whose ribs are abnormally brittle. That this injury is sometimes inflicted by the unjustifiable roughness of attendants may be admitted, that it seldom is so is my own experience. There were 13 instances of fractured ribs in the course of 25 years; of these three occurred during a struggle with attendants. None of the patients were paralytics, but, on the contrary, were strong, powerful men, and the attendants were acting in self-defence; one during a struggle with a fellow patient; three by being struck by a fellow patient; four accidentally from the patient falling on the floor, or on the sharp edge of a table or chair; and in three cases it was not ascertained how the injury occurred."

SCANDALS.—Our readers will be sorry, yet glad, to hear that the nurse of Selly Oak Workhouse, mentioned last week, has since been found insane, and removed to an asylum. But another case is now causing excitement. On December 5 Nurse Rose Morris, of Bolton Workhouse, died very suddenly. Recently she had brought grave charges against certain members of the board, and the Local Government Board had been petitioned in respect of these charges, Nurse Morris and Dr. Marsh, medical officer at the workhouse, being suspended. Sensational disclosures are expected.

SOMETHING NEW IN JOURNALISM.—A correct announcement appeared in several daily papers for November 29th of the forthcoming Exhibition of Nursing Unifoims, headed "Something New in Doll Shows." But this was too tame for an American exponent of the new journalism, who twisted about the paragraph, apparently with a desire to make it appear a bit of original news, until all accuracy was lost. This was how they stated it: "The nurses at the Charing Cross Hospital have for some time occupied their spare moments in preparing clothes for dolls and in dressing them. The dolls will be in sets, and will represent the uniform of the probationer, staff nurse, and sister of some hospital. An exhibition will be held at the hospital on Saturday week."

WHERE "THE HOSPITAL" CAN BE BOUGHT.—A correspondent wrote to us from Derby saying it would be a great boon to private nurses if THE HOSPITAL could be procured at any bookstall, as institutions do not forward papers to their private nurses. We were under the belief that our paper could be had at any bookstall, and made enquiries at Derby railway station and found it had a prominent position there. If our readers would only ask at the railway stalls, Messrs. W. H. Smith and Son assure us they would easily obtain THE HOSPITAL; but, in any case, the authorities of the nursing institutions might be at the trouble of forwarding papers for the amusement and instruction of their nurses, who are often cut off from the outer world by long attendance on infectious or trying cases.

AMERICAN NOTES.—We learn from a New York paper that the return of Miss Clara Barton, president of the Red Cross, from Johnstown, Pa., was celebrated by a reception got up by a number of leading men and women in Washington. The gathering was held at Willard's Hotel, and was very numerously attended. Commissioner Douglas and Judge McArthur proffered a warm welcome in the name of the people of the district, but the reports do not mention any ladies as having joined in words of homage to their brave sister.—Miss Vida Lathom, sister of Dr. Lathom, of Cambridge, is assistant in the pathological laboratory at Michigan.—The General Hospital at Ottawa is nursed by a sisterhood of grey nuns. It is capable of holding about fifty patients. There is also a Protestant hospital on Sandy Hill, which holds about thirty patients, and the old hospital is used for contagious diseases.—One of the best training schools for nurses in America is that connected with Rochester City Hospital, N.Y. Over six hundred patients are treated a year, including many confinement cases. The first graduation exercises of this school were held in 1883, when six pupils graduated with an elaborate programme of essays, music, etc. Over eighty lectures a year are given by the medical staff, and the nurses are instructed in massage and sick cookery. The course lasts three years; the age limit is twenty to thirty-five.

### The Show of Nurses' Uniforms.

ONCE some well-meaning person, anxious to prove that the girl graduates of Girton and Newnham were not wholly lost to the softer aspects of womanhood, announced proudly that they had held a doll show. Tiny dresses had been contrived by mathematic brains that dared to undergo the test of the tripos, and hands familiar with the pen had not disdained to use the needle. And everybody thought it was very much to these students' credit. It is hardly necessary at this time of day to prove that our nurses are women after all, even though they don't faint at the sight of blood and know how to use a clinical thermometer. But many people think that they must be rather sad women, living as they do in an atmosphere of sickness, sweet, perhaps, in a sentimental way—the ideal nurse of the outside public is always sentimental—but removed by the seriousness of their vocation from the pleasant frivolities of life. To think of a nurse dressing a doll!

Well, they have done it, and the result of their play-work, taken up when the labour of the ward was done, or the round of the district accomplished, will be shown to the public next Monday in the board-room of Charing Cross Hospital—300 doll-nurses, whose costumes accurately represent the uniforms worn by sister, staff-nurse, and probationer at 100 institutions. The three grades above mentioned are not in existence in all hospitals, but as a rule there is something corresponding to them, and very few of the competitors have failed to send in the required set of three dolls. The Editor of THE HOSPITAL is often asked by his correspondents about details of nursing uniform. Some who thus enquire are anxious to avoid infringing on their neighbours' rights; others are desirous of imitating the style of popular institutions. To both he now gives the same advice—go to Charing Cross Hospital on Monday (10 a.m. to 6 p.m.) or Tuesday (10 a.m. to 1 p.m.), the 9th and 10th inst., there see what is worn and so learn what to wear. It is intended, moreover, to keep specimens of the uniform worn in well-known hospitals and institutions, and thus to form a sort of museum of nursing dress. Prizes will be given for the best-dressed dolls—"best" meaning in this case accuracy of detail as wel as fineness of needlework, and the judges will be Miss Gordon, matron of Charing Cross Hospital; Miss Hicks, matron of the Hospital for Sick Children, Great Ormond-street; and the Editor of the NURSING MIRROR, supplement to THE HOSPITAL.

Of course, there is endless variety in the quality of the work, though all the specimens sent in show loving care and no little pains. "My doll's clothes come off and on," says one competitor. Another mentions an inaccuracy that could not be avoided. "'Fringes' are not allowed at our hospital, but I could not get dolls without them." It is well known that dolls are women of fashion to a much larger extent than nurses, and almost all these have their caps resting on rows of elaborate curls. But in almost all respects the strictest accuracy is followed. The streamers that hang from the sister's cap, the train that dignifies the matron's gown, are there to the life though in miniature. We cannot help wondering at the skill which fashioned the tiny pincushion, duly supplied with safety pins, and some trouble must have been expended in getting those microscopic chatelaines, whose scissors, as the popular song hath it, "won't cut; but they'll open and shut, and that's better than nothing at all." It is satisfactory to note that some careful sisters have their inkstand and pen with them ready for the doctor's tour of the ward. Several of the nurses have their casebooks in the pocket of their aprons, and a large number have bandages ready to hand; in fact, tied on. Realism is thus carried, it will be seen, to the furthest extent. Only in one point, if one may hint at this, does it break down—in the matter of "hosen and shoon." If any nurses wear such dainty lace stockings and bronze or patent leather boots—well, we don't know them, that's all. It may be noted, too, that some of the nurses, being of that variety of doll which opens or shuts its eyes according to the position in which it is held, show a reprehensible tendency to shut their eyes even when on duty. We hope that this does not point to a similar weakness on the part of their human prototype.

Without indulging in a catalogue, we may touch on some of the most characteristic uniforms. First—for we love "the services," as all England should—let us speak of the naval and military uniforms. The army sisters wear a grey gown with a scarlet cape, a white apron, and a rather fantastic white cap; while the naval ones are, of course, clad in blue, the short blue cape being enlivened with scarlet facings. The costume of the National Aid Society's nurses is similar to that of the army sisters, with the addition of a Maltese cross of white cloth upon the cape. The sisters at the London Hospital wear a gown of blue cashmere, and a lawn cap with long streamers. Sisters, almost everywhere, wear dresses of blue or brown cashmere or serge, though Great Ormond-street breaks the rule with a trained dress of dark prune colour. The nurses and probationers tend most to galatea, and print in small blue, pink, brown, or lilac stripe or check. Where more elaborate patterns are used the effect is seldom conspicuously happy. At Middlesex, again, however, the lady pupils wear black alpaca dresses, and a purple ribbon in their caps. At the Children's Hospital, Sheffield, the matron indulges in a dainty apron of blue check above a dark blue gown; and at St. Saviour's Hospital in Osnaburg-street the day nurse wears a red apron, but the all but invariable rule for aprons, whether they are of the finest lawn or the coarsest linen, is that they are as white as bleaching can make them. Of caps, who shall describe the variety—those of muslin and those of net, those with strings and those without, those with lace-edged borders and those severely plain. The materials of which these uniforms are made are few and simple, but the combinations are so numerous that it would be difficult to point to two dresses that are exactly the same. The cap alone gives scope for infinite shades of difference, and whoso will observe the length and width of aprons, and the shape of the bib, will admit that nursing uniforms are not quite so much "all the same" as he in his ignorance fancied.

But the daintiest uniform does not always denote the most pleasing work. This gown of light blue zephyr, with the holland apron whose fawn tint contrasts so well with it, raises a sigh of envy—till one sees that it is the dress of the fever nurses at the Norfolk and Norwich Hospital. The red and white stripe that is so popular in children's hospitals is charming, and the nursing of children has an attractive sound; but it is by no means the easiest part of the profession. The night nurses' grey tweed gowns are not picturesque, but they are warm and comfortable, which is, on the whole, more important. The nun-like dress of the sisters at University College Hospital is effective in its quaint severity, but it is the sign of a life that has given up earthly ambitions. This nurse in outdoor dress forms a graceful figure in her long blue cloak, under which you may catch a glimpse of a covered basket. That basket means district work, which means, as many can tell you, long weary trudges by muddy streets and unpaved country lanes in the bleak winter weather. There is not one of these simple dresses but speaks to those who know of watchful night and laborious days, of pleasures put aside, and work, hard, painful, often repulsive work, accepted in their stead, for the sake of the suffering and the poor.

This is what our doll show comes to after all. Something more than a collection of puppets, something more than a display of becoming, though most simple, dress—a reminder, small and lifeless, indeed, but still a reminder of the necessary, noble work that is done daily and nightly by the women of our land, and helps to mitigate the burden of humanity.

### The Nurses' Bookshelf.

### MASSO-THERAPEUTICS.*

Dr. Murrell is very hard on what passes during the present day as "massage"; his notion of massage is something far different. He says: "The so-called 'masseur' or 'masseuse,' who goes about the country armed with a certificate which is simply a receipt for money paid is an abomination, and has been the means of bringing a legitimate mode of treatment into disrepute. The confusion which still exists between massage and Weir-Mitchellism is also unfortunate." Nothing but the thorough system of Mezger and Von Mosengeil will satisfy Dr. Murrell. In this book massage is defined as a scientific mode of treating certain forms of disease by systematic manipulation. The chief manipulations are effleurage, a stroking movement made with the palm of the hand, passing with various degrees of force over the surface centripetally; pétrissage, picking up and pressing and rolling the muscles and tissue; friction, which is done with the tips of the fingers, and is employed chiefly on the joints; and tapotement, a kind of percussion made with the tips of the fingers or radial border of the hand. Dr. Murrell is in favour of two years' training for a masseuse, and says a certain amount of intellect and refinement are necessary to secure competence in the art. "Degenerate rubbing" is the term applied to the massage practised in many hospitals and institutions. A large number of specimen cases of paralysis, constipation, rheumatism, neurasthenia, and others are given, in which massage has proved beneficial, ending up with the case of the lady who had an inordinate craving for tobacco. Women smokers had better be careful if massage is to be the treatment for such of their sex as take to smoking. Dr. Murrell writes well, but somewhat flippantly, and seems to have a special grudge against marriage. He says: "Another lady, whose age was reputed to be thirty-five, had her waist reduced by massage from 25in. to 20in., and made an excellent marriage." Again, "People are not very particular to distinguish accurately between isolation and seclusion, and to say of a young lady that she has been in seclusion is not to improve her matrimonial prospects," and "as seriously ill as any respectable young married woman could wish to be." The following, also, is not exactly the most dignified style for a scientific treatise: "In that common domestic affliction, a black eye, massage, if properly performed, will remove all traces of the disagreement in three days. This is better than the plan usually recommended of painting the other eye in order to ensure uniformity."

Dr. Murrell makes ample acknowledgment to the fellow-physicians who have aided him, and places an excellent index at the end of his book.

### OUR TOILING SISTERS.†

This is a book in which we can trace the hand of the lady who chooses to be known as "John Law"; the truthful pathetic pictures could have come from no other pen. The book is one of interest to all women, and especially to all nurses who would sympathise with and understand the lives of their patients. It is an enquiry into female labour in the metropolis, and deals with match-girls, flower-girls, brush-makers, slaveys, barmaids, etc. Vivid pictures of the awful poverty which lies close to the gross luxury of the metropolis are shown to us, and ought to raise in every reader's heart not only the longing, but the firm resolve to seek to aid these our poorer sisters. A hearthbrush with 109 holes has the fibres put in the holes and firmly wired at ½d. a brush. "They take a lot of time, and they're that troublesome I'd never do 'em but for mother," said the girl who worked all the day and half the night at these brushes. Poverty and sin, hunger and dirt, and slavery more degrading than that of any Eastern country. And only by women's work for women are these evils being conquered. May this book fall into many hands—touch many hearts.

* "Masso-Therapeutics; or, Massage as a Mode of Treatment." Fourth Edition. By William Murrell, M.D., F.R.C.P. H. K. Lewis.

† "Toilers in London." By the *British Weekly* Commissioners. Hodder and Stoughton.

### Everybody's Opinion.

*[Correspondence on all subjects is invited, but we cannot in any way be responsible for the opinions expressed by our correspondents. No communications can be entertained if the name and address of the correspondent is not given, or unless one side of the paper only be written on.]*

### A FIRST EXPERIENCE.

Nurse Freddy writes from New Jersey: I read your paper with the greatest pleasure; every word seems to interest me, and I have thought that an account of my first operation might interest some of your readers, for few I trust have had such a sad experience. It happened years ago; if not I do not think I could have written it, for even now the tears will come as I think of all that happened. To see an operation for the first time must, I think, always cause considerable anxiety and nervousness even to the most callous and self-possessed. Fortunate are those nurses who have their first experience in the well-regulated wards or theatre of an English hospital, where they are surrounded by responsible people, and furnished with all the modern improvements to save time and alleviate pain. The new probationer is often as anxious about doing her own work (washing sponges) well as about the operation itself. I saw my first operation under very different circumstances. I can picture the scene now. A large, barely furnished room, the gas flaring (for it was midnight), two doctors in spectacles whispering in a strange tongue, a golden-haired frightened baby cling round the neck of a young girl, who, pale with fear herself, tried to kiss and comfort him. Poor little soul! I had loved and petted him, and now, with the innocent trust of childhood, he nestled in my arms to be taken care of. An assistant was preparing the table, etc. The mother and all the other women in the house had sought refuge as far away from the scene as possible. I begged one of them to stay with me, but they were too nervous. Fancy leaving that pretty baby alone with strange men! At last all was ready, and I must lay him down, but the doctor had assured me that it would not be much, and that baby was sure to live through it. So I kissed my pet, and told him to be a good boy, and I would give him lots of pretty things to-morrow. I unclasped his tiny fingers, and laid bare his little white neck and chest, he calling me by name all the time between the gasps of laboured breath. (It was croup he was suffering from, and tracheotomy was to be performed.) I had to hold his feet, and the assistant held his hands, for they gave him no anæsthetic. Now commenced the most trying time of my life. I had to keep my voice steady so as to talk to baby to soothe him, whilst I held his little struggling limbs in a vice. At the same time I was trying to catch what the doctors were saying to each other, for their low guttural German voices, using medical terms, would have puzzled me at any time. I kept my face turned away most of the time, and tried to pray; but oh, it seemed so long to wait! Ten, fifteen, twenty minutes passed, then a shiver passed over the little frame, the struggling limbs lay quite still, never to move again. I only thought he had fainted; I waited, I dared not stir, at least, I heard one doctor say to the other, "That has gone wrong." Then I looked up; oh, what a sight met my frightened gaze! Baby was black in the face. There was a gaping wound at least an inch long in his throat; his golden curls were matted in blood, which also drenched the sheets and floor. The doctors did not speak to me, but began at once to wipe their knives. I asked, in a voice choking with fear and grief, "Is the child dead?" They answered, "It happened so." I felt at that moment I could have killed them. I was almost mad. How I got baby into his cot, and cleared the room and told the mother, I hardly know.

CO-OPERATIVE REGISTRY FOR NURSES.

ONE OF MANY writes: We should like "Not in Want of Work" to know that the registry was suggested, not only to fulfil what seems a great need, but as the nucleus of a nurses' union, with homes and other benefits attached. The confidence of the public would be secured by a committee, either of ladies and medical men, or of honorary members—matrons, sisters, etc.; this confidence must be further enhanced by our choice of a superintendent. Those of our number who can be spared from patients will be glad to meet "Not in Want of Work" and her friends on the day fixed by the Hospitals Association, and we trust that we shall be able to combine for the advantage of all.

### Notices.

EXHIBITION OF NURSING UNIFORMS.—The exhibition will be open at Charing Cross Hospital on Monday, December 9th, from 10 a.m. to 8 p.m., and on Tuesday, December 10th, from 11 a.m. to 6 p.m. All nurses in in or outdoor uniform and all exhibitors will be admitted free. The public will be charged 1s. each. The names of the prize winners will be announced next week; should any prize winner prefer books to money, will she please write and say so.

EXAMINATION QUESTIONS.—In consequence of the large number of answers received and the extra work caused by the doll show, the Editor is unable to announce this week which is the best answer. All particulars will appear next week.

### Fair Dispensers.

[Some ladies—Misses Brittain, Moore, Cook, and Bowen—have recently qualified and been appointed dispensers at various hospitals.]

Here are ladies as dispensers, each a fascinating fixture
In the surgery, presiding over measures, weights, and scales;
They will spread a careful blister, or will make you up a mixture,
That should surely prove more potent than the same dispensed by males.
They've forsworn the fads of fashion wearing ermine or chinchilla,
But they understand prescriptions and the doctor's mystic sign;
Does your system need a tonic, there the pleasant *Cascarilla*,
And the piquant *Tinct. Aurantii*, like bitters ere you dine.

You will never shirk your physic when a maiden wields the measures,
And drops in the merry minim, or the calculated drachm;
You will rank her mustard-plaster 'mid the first of earthly pleasures,
And you'll take her pretty powders without asking for the jam.
When a fairy hand compounds it, sure a dainty draught or potion
Should possess a unique value for the cure of human ills;
You will rub it in quite gaily when a lady makes a lotion,
And will even, pray believe me, find there's poetry in pills.

Every bottle when it's opened will be sweet as any posy,
And you'd like to take more doses than the label says is right;
For they'll tempt you on occasion with the sweet *Syrupus Rosæ*.
And the charming *Tinct. Lavandulæ* will flash upon your sight.
Give the ladies, then, a bouquet of the *Aloe Barbadensis*,
With the *Folia Digitalis* crown the pharmaceutic *belle*;
Fill the goblet *Vino Xerici* to each one who dispenses,
May they never need the physic they themselves make up so well.

*St. James's Gazette.* H. SAVILE CLARKE.

### Notes and Queries.

TO CORRESPONDENTS.—1. Questions may be written on post-cards. 2. Advertisements in disguise are inadmissible. 3. In answering a query please quote the number. 4. If a private answer is desired, a stamped addressed envelope must be forwarded.

NOTICE.—We must really ask our correspondents to be more particular in enclosing name and address. Constantly we receive queries or letters with no name attached.

### Queries.

*Massage.*—Will someone kindly say where massage can be thoroughly learnt in Bristol; also in London? What is the fee for the course? And if slight deafness would be any hindrance in the work? *Nurse Vivien.*

*Dispensing.*—Where can a nurse learn dispensing, and for what fees? West-end of London preferred.

### Answers.

*Marjorie.*—Seeing your advertisement in THE HOSPITAL this week, I write to tell you that if you write to Mrs. Perrins, St. John's Convalescent Home, North Malvern, I think you will have a very satisfactory answer. *Nurse J. S.*

### Presentations.

LAMBETH INFIRMARY.—On Thursday, the 21st of November, the nurses and officers of the Lambeth Infirmary presented Dr. Grosvenor Shaw, the assistant medical officer, with a very handsome cut glass and plate sideboard piece. Handsome as the gift was, much more so were the kind and appreciative feelings which prompted the presentation.

MONSALL FEVER HOSPITAL.—Miss E. Ross, lady superintendent of nurses, who is leaving for the Western Hospital, Fulham, has been presented with a very handsome dressing-case by the nursing staff of this excellent hospital.

### Vacancies.

THE following vacancies are announced:

### Matrons.

Clifton College Sanatorium.
Dunster and Minehead Village Hospital.
Longton Cottage Hospital, £50.
Sheffield Union (assistant), £35.
West of England Eye Infirmary, £40.

### Sisters.

Swansea Hospital (two), £25.

### Nurses.

Barrow-in-Furness Hospital, £20.
Bedfordshire Institute, £26.
Birmingham Hospital for Women, £23.
Blackheath Institute, £20.
Canterbury Nurses' Institute.
Carlisle Infirmary.
Central London Throat Hospital, W.C., £20.
Chester Deaconess Institute, £24.
Chichester Infirmary, £26.
Devon and Exeter Hospital, £24.
Eastbourne Institution.
Frome Nurses' Home, £22.
Hampstead Home Hospital, £20.
Highbury Institute.
Jersey Nurses' Institute (two), £25.
Kensington Infirmary, £20.
King's Cross Hospital, Dundee, £24.
Leicester Institution, £25.
Liverpool City Hospital (two), £25.
Liverpool Foundling Hospital.
Newcastle City Hospital.
Newport Institute.
Nursing Institution, 96, 97, 98, Wimpole-street, London, W., Mr. Wilson has several vacancies for thoroughly trained nurses.
Paddington Children's Hospital.
Peterborough Infirmary, £20.
Royal Albert Hospital, Devonport.
Royal Cornwall Infirmary, £20.
Ryde and Ventnor Homes.
Sheffield Nurses' Home.
Southport Infirmary (night), £20.
Southport Nurses' Home.
Southport, St. John's Institute, £25.
St. Helena Home, N.W. (several).
St. Leonard's Home.
Stratford-on-Avon Hospital, £20.
Swansea Nurses' Institute (several), £30.
West London Hospital, £28.
Western Fever Hospital (several), £22.
Woking Industrial School, £24.
Wolverhampton Institution.

### District Nurses.

Queen Victoria Institute, Edinburgh
Whalley Range, Manchester, £60.
Yarm, Yorkshire, £60.

### Probationers.

Barrow-in-Furness Hospital.
Canterbury Hospital.
Central London Ophthalmic Hospital.
St. Helens Cottage Hospital.
Swansea General Hospital.
Wolverhampton Institution.
West London Hospital.

### Amusements and Relaxation.

**Third Quarterly Word Competition Commenced October 5th, 1889, ends December 28th, 1889.**

Three Prizes of 15s., 10s., 5s., will be given for the largest number of words derived from the words set for dissection.

Proper names, abbreviations, foreign words, words of less than four letters, and repetitions are barred; plurals, and past and present participles of verbs, are allowed. Nuttall's *Standard* dictionary only to be used.

N.B.—Word dissections must be sent in WEEKLY, arranged alphabetically, with correct total affixed.

The word for dissection for this, the *Tenth* week of the quarter, being "P L O V E R S."

### Results of Eighth Week.

| Names. | Nov. 28th. | Totals. | Names. | Nov. 28th. | Totals. |
|---|---|---|---|---|---|
| Winifred | — | 672 | Sunshine | 220 | 1127 |
| Adelaide | — | 646 | Electrician | 236 | 1268 |
| Nettie Vance | — | 757 | H. C. | — | 562 |
| Amos | — | 596 | Reldas | 253 | 1328 |
| F. W. P. H. | 254 | 1314 | Rover | — | 776 |
| Reyot | — | 369 | Qu'appelle | 187 | 1073 |
| Ellice | 256 | 1345 | Beryl | — | 188 |
| A. S. K. | — | 275 | Lightowlers | 248 | 1370 |
| Neptune | 199 | 1254 | Hercules | — | 12 |
| Ecila | 252 | 1401 | Jenny Wren | 210 | 940 |
| Tinie | — | 17 | Night Watch | — | 346 |
| Amicus | — | 946 | Oakenrod | — | 1122 |
| Juvenis | — | 539 | Embryo | — | 385 |
| Jumps | 241 | 1395 | Gypsye | — | 967 |
| Sister | — | 438 | Leo | — | 676 |
| M. W. | 226 | 1338 | Pearl | — | 299 |
| Esperance | 233 | 1337 | Africando | — | 171 |

